# The short physical performance battery and incident heart failure among older women: the OPACH study

**DOI:** 10.1016/j.ajpc.2021.100247

**Published:** 2021-08-20

**Authors:** John Bellettiere, Steve Nguyen, Charles B. Eaton, Sandy Liles, Deepika Laddu-Patel, Chongzhi Di, Marcia L. Stefanick, Andrea Z. LaCroix, Michael J. LaMonte

**Affiliations:** a Herbert Wertheim School of Public Health and Human Longevity Science, University of California San Diego, La Jolla CA, USA; b Center for Behavioral Epidemiology and Community Health (C-BEACH), School of Public Health, San Diego State University, San Diego, CA, USA; c Departments of Family Medicine and Epidemiology, Schools of Medicine and Public Health, Brown University, Providence, RI, USA; d College of Applied Health Sciences, University of Illinois, Chicago, IL, USA; e Division of Public Health Sciences, Fred Hutchinson Cancer Research Center, Seattle, WA, USA; f Stanford Prevention Research Center, Department of Medicine, Stanford University School of Medicine, Stanford, CA, USA; g Department of Epidemiology and Environmental Health, School of Public Health and Health Professions, University at Buffalo, SUNY, Buffalo, NY, USA

**Keywords:** Geriatric cardiology, Physical functioning, Healthy cardiovascular aging, Frailty

## Abstract

**Objective:**

Reduced functional capacity is a hallmark of early pre-clinical stages of heart failure (HF). The Short Physical Performance Battery (SPPB) is a valid measure of lower extremity physical function, has relatively low implementation burden, and is associated with cardiovascular disease and mortality. However, the SPPB-HF association is understudied in older women among whom HF burden is high.

**Methods:**

Women (*n* = 5325; mean age 79 ± 7 years; 34% Black, 18% Hispanic, and 49% White) without prior HF completed the SPPB consisting of standing balance, strength, and walking tests that were summarized as a composite score from 0 (lowest) to 12 (highest), categorized as very low (0–3), low (4–6), medium (7–9), or high (10–12). Participants were followed for up to 8 years for incident HF (306 cases identified). Cox proportional hazards regression estimated hazard ratios (HR) adjusting for age, race/ethnicity, education, smoking, alcohol, diabetes, hypertension, COPD, osteoarthritis, depression, BMI, systolic blood pressure, lipids, glucose, and accelerometer-measured moderate-vigorous physical activity (MVPA) and sedentary time.

**Results:**

Incident HF cases (crude rate per 1000 person-years) in the four SPPB categories (very low to high) were 34 (26.0), 79 (14.5), 128 (9.3), and 65 (5.6). Corresponding multivariable-adjusted HRs (95% CIs) were 2.22 (1.34–3.66), 1.63 (1.11–2.38), 1.39 (1.00–1.94), and 1.00 (referent; *P*-trend<0.001). Higher HF risk was associated with lower SPPB in women with major modifiable HF risk factors including obesity (HR per 3-unit SPPB decrement: present HR = 1.41, absent HR = 1.41), hypertension (present HR = 1.45, absent HR = 1.30), diabetes (present HR = 1.32, absent HR = 1.44), and lower accelerometer-measured MVPA (<45 min/day HR = 1.29, ≥45 min/day HR = 1.60); all *P*-interaction>0.10.

**Conclusion:**

Lower SPPB scores were associated with greater risk of incident HF in older women even after accounting for differences in HF risk factors and objectively measured PA. Implementing the SPPB in clinical settings could potentially enhance individual-level HF risk assessment, which should be further explored.

## Highlights

• In older women, lower Short Physical Performance Battery (SPPB) scores were associated with higher incident heart failure (HF) risk.
• SPPB-HF associations were consistent in women with obesity, diabetes, hypertension, and low physical activity.
• Including the SPPB routinely in clinical evaluations could enhance HF prevention.

## Introduction

Heart failure is a major public health challenge that increases morbidity, mortality, and healthcare costs [1]. Heart failure prevalence is estimated to be 3.9% in women aged 60-79 and 11.0% in women aged 80 or older [1]. From 2011–2017, deaths from heart failure increased by 38%, and the estimated lifetime risk of heart failure from age 45 to 95 was 32–39% in White women and 24–46% in Black women [1,2]. Improved knowledge of heart failure risk factors could support efforts for individual-level risk stratification and population-level prevention.

Low physical function is an early manifestation of preclinical heart failure, due to impaired myocardial energetics and skeletal muscle myopathy that alters myofibrillar contractile properties [3]. The SPPB is an objective, performance-based test of lower extremity function that consists of chair stand, standing balance, and timed walk tests designed to be quickly administered without specialized equipment [4]. Higher SPPB scores have been consistently associated with lower risks of all-cause and cardiovascular mortality and with incident cardiovascular disease events [5,6]. The extent to which SPPB is related to incident heart failure is understudied [7]. Additionally, there have been relatively few studies in racially/ethnically diverse older women, among whom heart failure risk is high [1].

In the present study, we examined whether higher SPPB scores were associated with lower risk of incident heart failure among women aged 63–99 years with no history of heart failure who were followed for up to 8 years. We additionally examined whether the SPPB-heart failure association differed by age, BMI, cardiovascular disease risk based on the Reynolds Risk Score, physical activity, diabetes, hypertension, and race/ethnicity.

## Methods

### Study population

The Women's Health Initiative (WHI) is an ongoing study of the determinants of morbidity and mortality in aging postmenopausal women. WHI enrolled 161,808 postmenopausal women, aged 50–79 years, into one of three randomized clinical trials or a prospective observational study across 40 US study sites from 1993 to 1998 [8]. During the WHI 2010-2015 extension study, home examinations including the SPPB were completed in a sub-cohort of 7,875 women also enrolled into the ancillary Long Life Study (2012-2013). The Objective Physical Activity and Cardiovascular Health (OPACH) Study was ancillary to the Long Life Study and consisted of 7,058 ambulatory community-dwelling women aged 63–99 years who were recruited to complete an accelerometer study aimed at measuring free-living physical activity and sedentary behavior between May 2012 to April 2014, and relate these measures with cardiovascular disease incidence during follow up through February 28, 2020 [9]. Of the 6,489 women who returned accelerometers, 6,382 had data for at least 1 adherent day using the common definition of at least 10 hours of accelerometer wear time while awake. Of these, we excluded data from 903 women who did not have SPPB measured and 154 who had heart failure prior to SPPB measurement, resulting in an analytic sample of 5325 women who were followed for incident heart failure. Study protocols were approved by the Fred Hutchinson Cancer Research Center (WHI Coordinating Center) and all women provided informed consent in writing or by phone.

### Incident acute decompensated hospitalized heart failure

Incident acute decompensated hospitalized heart failure was ascertained annually in WHI by medical record abstraction of self-reported hospitalizations and classified by trained physician adjudicators using standardized methodology [10,11]. Acute decompensated hospitalized heart failure requiring and or occurring during hospitalization required physician diagnosis of new onset or worsened congestive heart failure on the reported hospital admission and at least 1 of the following 4 criteria: (1) physician-diagnosis and medical treatment for heart failure, (2) symptoms and documentation in the medical record of a history of an imaging procedure showing impaired left ventricular (LV) systolic or diastolic LV function, (3) pulmonary edema or congestion on chest x-ray on admission, or (4) “poor” LV or right ventricular function by echocardiography, radionuclide ventriculography, or other contrast ventriculography or evidence of LV diastolic dysfunction [10]. The agreement between central adjudication and local adjudication was high with a Kappa of 0.79 [11].

### Short physical performance battery (SPPB)

The SPPB measures lower extremity function and consists of 3 tests: time to complete 5 unassisted chair stands, 3 progressively more difficult standing balance tests that are each held for 10 seconds, and a 4-meter usual-paced walk to assess gait speed [12]. Each test is scored from 0 (lowest) to 4 (highest) using previously defined and validated cut-points which were then summed for an overall score ranging from 0 to 12, with higher scores indicating higher physical function. Previously defined categories were used for analyses: very low (0–3), low (4–6), moderate (7–9), and high (10–12) [5].

### Physical activity and sedentary behavior measurement

OPACH women wore an ActiGraph GT3X+ triaxial accelerometer over the right hip 24 h-per-day for up to 7 days except when they risked submerging the accelerometer in water (e.g., during bathing or swimming). ActiLife version 6 software was used to aggregate 30 Hz data into 15 s epochs. The Choi algorithm identified periods of accelerometer non-wear [9,13]. Sleep time was identified using self-reported in-bed and out-of-bed times from sleep diaries that were filled out each night of accelerometer wear. We then applied vector magnitude-based cut-points determined in a separate intensive calibration study among 200 OPACH women to define moderate-vigorous physical activity (MVPA; >518 counts per 15 s) and sedentary time (≤18 counts per 15 s) [14]. We used the residuals method to adjust MVPA and sedentary time for awake wear-time [15].

### Covariates

Questionnaires ascertained participant age, race/ethnicity (Black, White, Hispanic/Latina), educational attainment (high school equivalent or lower, some college, college graduate), alcohol consumption in the 3 months prior to baseline (non-drinker, less than 1 drink per week, 1 or more drinks per week, unknown), current smoking status (yes or no), and history of chronic obstructive pulmonary disease (COPD), osteoarthritis, and depression. Participants reported history of physician diagnoses for diabetes or hypertension treated with medications prior to OPACH baseline. During the Long Life Study home examinations, trained study staff measured height (m) and weight (kg) with a tape measure and calibrated scale and calculated body mass index (BMI; kg/m^2^). An aneroid sphygmomanometer was used to measure systolic and diastolic blood pressure, and the average of 2 measurements was recorded. Fasting (12 h) blood samples were obtained and serum glucose, total cholesterol, high density lipoprotein (HDL) cholesterol, triglycerides, creatinine, and high sensitivity C-reactive protein (CRP) were measured using standardized Clinical Laboratory Improvement Act-approved methods at the University of Minnesota [16]. Estimated glomerular filtration rate (eGFR) was calculated using the Chronic Kidney Disease Epidemiology Collaboration (CKD-EPI) equation using serum creatinine, age, sex, and Black versus non-Black race [17,18]. We calculated the Reynolds Risk Score, a summary measure of one's prior probability of developing clinical CVD, using age, systolic blood pressure, total and HDL cholesterol, CRP, diabetes status, smoking status, and family history of myocardial infarction (MI) [19].

### Statistical analysis

We carried out all statistical analyses in RStudio 1.3.959 (https://rstudio.com/). We calculated means and standard deviations, or counts and proportions, for participant characteristics and compared these values by SPPB categories at baseline using F-tests for continuous variables and chi-square tests for categorical variables.

Multivariable Cox proportional hazards regression models estimated hazard ratios (HRs) and 95% confidence intervals (CI) for the associations of SPPB categories with incident acute decompensated hospitalized heart failure. We defined follow-up time as the number of days from OPACH baseline to the first occurrence of an acute decompensated hospitalized heart failure, or date of the last obtained annual medical update. Schoenfeld residuals were used to test the proportional hazards assumption; no violations were noted. Cox regression models were progressively controlled for potential confounding factors. Model 1 adjusted for age and race/ethnicity. Model 2 additionally adjusted for educational attainment, alcohol consumption, smoking status, diabetes, hypertension, COPD, osteoarthritis, and depression. Model 3 contained all covariates that we considered confounders: model 2 covariates plus BMI. Models 4 and 5 additionally adjust for variables that could be considered mediators and or confounders. Model 4 contained model 3 covariates plus MVPA and sedentary time. Model 5 contained model 4 covariates plus systolic blood pressure, fasting serum glucose, HDL, and triglycerides. Because triglyceride concentrations were heavily skewed, all analyses were completed using log_10_ transformation, whereas untransformed concentrations in conventional units are reported descriptively. We imputed missing data for covariates using multiple imputation by chained equations (MICE) with the *mice* package in R, including all variables for 5 iterations and 100 imputations. We tested the linearity of the continuous SPPB-acute decompensated hospitalized heart failure association using restricted cubic splines with 3 knots at the 10^th^, 50^th^, and 90^th^ percentiles using the *rms* package in R and computing a Wald test for the nonlinear spline components. We carried out stratified analyses to determine consistency of the SPPB-acute decompensated hospitalized heart failure association within cohort subgroups defined by age (<80 years, ≥80 years), BMI (<30 kg/m^2^, ≥30 kg/m^2^), Reynolds Risk Score (<9.9, ≥9.9; median split), MVPA (<45 min/day, ≥45 min/day; median split), diabetes, hypertension, and race/ethnicity (Black, White, Hispanic/Latina). Here, HRs were estimated for a 3-unit decrement in SPPB, equivalent to one interquartile range, adjusted for model 3 covariates because covariates included in models 4 and 5 are likely on the causal pathway. We estimated models with cross-product interaction terms between each stratifying covariate and continuous SPPB to statistically evaluate effect modification. We set statistical significance for effect modification tests to *p* < 0.10 and *p* < 0.05 for all other tests.

For sensitivity analyses, since CVD can influence both SPPB and heart failure, we repeated analyses after excluding data from women who had prevalent CHD or stroke at the time of SPPB measurement. To reduce reverse causality bias, we also repeated analyses by excluding acute decompensated hospitalized heart failure cases that occurred within the first 6 months of follow-up. To investigate whether one of the three SPPB component tests (chair stands, balance, or gait speed) drove the SPPB-heart failure association, we calculated the Pearson correlation coefficient between each pair of the three tests; then a multivariable Cox regression (model 3 covariates) was performed for each component test included separately (modeled as a continuous variable ranging from 0 to 4), then a final model was estimated that included all three tests as independent variables.

## Results

### Study population characteristics

The average SPPB score was 8.3 with a standard deviation of 2.5 and approximately 77% of OPACH women had a SPPB score of 7 or greater indicating moderate and higher levels of physical function (Table 1). Compared to women with very low SPPB scores (0–3), those with high SPPB scores (10–12) were younger, were more likely to be of Hispanic/Latina ethnicity, were more likely to be a college graduate, were less likely to consume alcohol, had a lower BMI, were more likely to report excellent or very good health, and were less likely to have a history of treated diabetes or hypertension, COPD, osteoarthritis, or depression. Cardiovascular risk factor profiles were more favorable in women with higher SPPB scores. Those with SPPB scores between 10 and 12 had lower serum glucose and systolic blood pressure, higher HDL, and a lower 10-year predicted probability of a CVD event based on the Reynolds Risk Score as compared with women whose SPPB score was 0–3. Accelerometer-measured total sedentary time was lower, and light intensity physical activity and MVPA were higher, across incremental categories of SPPB (Table 1). Pearson correlations between the chair stand and balance test, chair stand and gait speed test, and balance and gait speed test were *r* = 0.22, 0.34, and 0.25, respectively.

**Table 1:** Participant Characteristics by SPPB Categories from the OPACH Study Baseline (2012-2014, *n* = 5325).

	SPPB Category				
	Very low (SPPB: 0–3)	Low (SPPB: 4–6)	Moderate (SPPB: 7–9)	High (SPPB: 10–12)	*p*-value
	*n* = 260	*n* = 955	*n* = 2273	*n* = 1837	
**Demographics**					
Age in years, *mean* (*sd*)	82.9 (6.3)	81.0 (6.2)	78.5 (6.5)	76.8 (6.5)	<0.001
Race/ethnicity*, n (%)*					<0.001
White	152 (58.5)	503 (52.7)	1065 (46.9)	867 (47.2)	
Black	84 (32.3)	328 (34.3)	861 (37.9)	533 (29.0)	
Hispanic/Latina	24 (9.2)	124 (13.0)	347 (15.3)	437 (23.8)	
Highest education level*, n (%)*					<0.001
High school or less	55 (21.4)	236 (24.9)	442 (19.6)	334 (18.3)	
Some college	109 (42.4)	366 (38.6)	856 (37.9)	701 (38.3)	
College graduate	93 (36.2)	345 (36.4)	962 (42.6)	793 (43.4)	
**Health behavior/status***, n (%)*					
Current smoker	4 (1.5)	25 (2.6)	69 (3.0)	39 (2.1)	0.209
Alcohol intake in past 3 months					<0.001
Non-drinker	109 (41.9)	397 (41.6)	798 (35.1)	509 (27.7)	
Less than 1 drink per week	68 (26.2)	271 (28.4)	716 (31.5)	603 (32.8)	
1 or more drinks per week	44 (16.9)	179 (18.7)	580 (25.5)	585 (31.8)	
Unknown	39 (15)	108 (11.3)	179 (7.9)	140 (7.6)	
Body mass index (kg/m^2^), *mean (sd)*	29.6 (7.2)	28.9 (6.3)	28.9 (5.6)	27.3 (5.3)	<0.001
Self-rated health					<0.001
Excellent or very good	75 (29.4)	339 (35.7)	1105 (48.8)	1203 (65.6)	
Good	115 (45.1)	448 (47.2)	969 (42.8)	562 (30.7)	
Fair or poor	65 (25.5)	162 (17.1)	191 (8.4)	68 (3.7)	
History of treated diabetes	72 (27.7)	251 (26.3)	493 (21.7)	267 (14.5)	<0.001
History of treated hypertension	219 (84.2)	751 (78.6)	1652 (72.7)	1182 (64.3)	<0.001
History of COPD	15 (5.8)	29 (3.0)	68 (3.0)	36 (2.0)	0.003
History of osteoarthritis	165 (63.5)	595 (62.3)	1250 (55)	907 (49.4)	<0.001
History of depression	37 (14.2)	99 (10.4)	171 (7.5)	127 (6.9)	<0.001
History of CHD	23 (8.8)	45 (4.7)	105 (4.6)	53 (2.9)	<0.001
History of stroke	14 (5.4)	50 (5.2)	70 (3.1)	37 (2.0)	<0.001
**CVD biomarkers**, *mean (sd)*					
Reynolds Risk Score*	19.0 (13.3)	17.1 (12.7)	13.3 (10.8)	10.9 (10.3)	<0.001
Systolic blood pressure (mmHg)	127.8 (16.2)	127.3 (14.3)	125.9 (14.1)	124.6 (13.7)	<0.001
Glucose (mg/dL)	100.8 (31.2)	100.6 (29.2)	97.4 (25.4)	96.2 (22.3)	<0.001
Total cholesterol (mg/dL)	189.0 (39.2)	195.1 (38.8)	197.0 (38.7)	202.4 (39.1)	<0.001
HDL cholesterol (mg/dL)	59.0 (15.0)	59.9 (14.5)	60.3 (14.8)	61.8 (15.2)	0.003
Triglycerides^b^	109 (56.9)	110 (53.6)	108.1 (55.9)	107 (54.0)	0.442
Log hs-CRP (mg/L)	0.8 (1.1)	0.7 (1.1)	0.6 (1)	0.5 (1)	<0.001
Creatinine (mg/dL)	0.9 (0.3)	0.9 (0.4)	0.9 (0.3)	0.8 (0.2)	<0.001
eGFR (ml/min/1.73m^2^)	64.2 (17.5)	66.0 (18.9)	70.1 (17.3)	72.9 (16.1)	<0.001
**Physical activity**^a^, *mean (sd)*					
Total sedentary time (hr/day)	10.3 (1.5)	9.6 (1.4)	9.2 (1.4)	8.8 (1.5)	<0.001
MVPA (hr/day)	0.5 (0.4)	0.6 (0.5)	0.8 (0.5)	1.1 (0.6)	<0.001
Light physical activity (hr/day)	4 (1.2)	4.5 (1.2)	4.8 (1.2)	5 (1.2)	<0.001

Abbreviations: SPPB = Short Physical Performance Battery; SD = Standard Deviation; COPD = Chronic Obstructive Pulmonary Disease; CHD = coronary heart disease; eGFR = estimated glomerular filtration rate; hr = Hour; MVPA = moderate-to-vigorous physical activity; hs-CRP = high sensitivity C-reactive protein.

p-value: Chi-square test for categorical variables and F-test for continuous variables.

*10-Year predicted probability (%) of a clinical CVD event.

a All physical activity-related variables are adjusted for accelerometer awake wear time using the residuals method.

b p-value calculated using log-transformed triglycerides to account for skewness

### Associations of SPPB with incident acute decompensated hospitalized heart failure

**Fig. 1:**
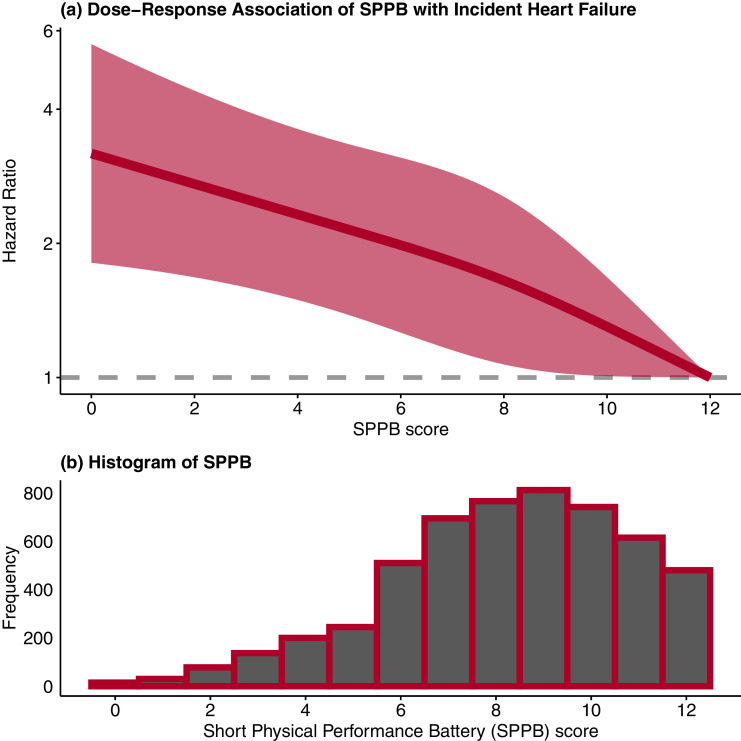
Dose-Response Association of Short Physical Performance Battery (SPPB) Score with Incident Acute Decompensated Hospitalized Heart Failure (Panel a) and Histogram for SPPB (Panel b). Results for the dose-response association were estimated using restricted cubic splines with 3 knots placed at 10th, 50th, and 90th percentiles, in models adjusting for age, race/ethnicity, education, alcohol, smoking, diabetes, hypertension, COPD, osteoarthritis, depression, and BMI. The reference group was an SPPB score of 12.

Over a median follow-up period of 6.5 years, there were 306 incident acute decompensated hospitalized heart failure events. The number of events (crude incidence rate per 1000 person-years) for acute decompensated hospitalized heart failure across very low (0–3), low (4–6), moderate (7–9), and high (10–12) SPPB scores were 34 (26.0), 79 (14.5), 128 (9.3), and 65 (5.6) person-years, respectively (Table 2). Significant inverse associations between SPPB category and incident heart failure were observed in multivariable Cox regression analysis. The HR (95% CI) for the associations with acute decompensated hospitalized heart failure risk according to very low, low, and moderate compared to high (referent) SPPB adjusted for age and race/ethnicity were 3.19 (2.07–4.89), 2.01 (1.44–2.82), and 1.50 (1.11–2.03), respectively, with *P*-trend<0.001 (model 1, Table 2). The inverse linear trends persisted after progressive adjustment for covariates including MVPA, sedentary time, and cardiovascular biomarkers across models 2–5 (*P* < 0.001, all) with some attenuation of the HRs observed. Results of restricted cubic spline regression analysis indicated that the continuous SPPB-heart failure dose-response association was linear (*p*-linear <0.001, *p*-non-linear=0.57; Fig. 1). When analyzed in continuous format (model 5 covariates), the HR (95% CI) for incident acute decompensated hospitalized heart failure associated with a one standard deviation (SD_SPPB_ = 2.5) unit decrement in SPPB score was 1.27 (1.12–1.44).

**Table 2:** Associations of Short Physical Performance Battery (SPPB) Categories with Incident Acute Decompensated Hospitalized Heart Failure in 5325 OPACH Women (2012–2020).

	SPPB Categories				
	Very Low (SPPB: 0–3)	Low (SPPB: 4–6)	Moderate (SPPB: 7–9)	High (SPPB: 10–12)	*p*-trend^a^
	*n* = 260	*n* = 955	*n* = 2273	*n* = 1837	
Events [rateb]	34 [26.0]	79 [14.5]	128 [9.3]	65 [5.6]	
Model 1^c^	3.19 (2.07-4.89)	2.01 (1.44-2.82)	1.50 (1.11-2.03)	1 (ref)	<0.001
Model 2^c^	2.67 (1.73-4.13)	1.79 (1.27-2.52)	1.41 (1.04-1.91)	1 (ref)	<0.001
Model 3^c^	2.59 (1.67-4.04)	1.78 (1.26-2.51)	1.39 (1.03-1.90)	1 (ref)	<0.001
Model 4^c^	2.21 (1.41-3.47)	1.58 (1.11-2.24)	1.30 (0.95-1.77)	1 (ref)	<0.001
Model 5A^c^	2.22 (1.34-3.66)	1.63 (1.11-2.38)	1.39 (1.00-1.94)	1 (ref)	<0.001
Model 5B^d^	2.10 (1.34-3.30)	1.54 (1.09-2.18)	1.28 (0.94-1.74)	1 (ref)	<0.001

Model 1 is age and race/ethnicity adjusted [*n* = 5325].

Model 2 = Model 1 + education + smoking status + alcohol use + diabetes + hypertension + COPD + osteoarthritis + depression [*n* = 5292].

Model 3 = Model 2 + BMI [n=5253].

Model 4 = Model 3 + sedentary time + moderate-to-vigorous physical activity [*n* = 5253].

Model 5 = Model 4 + systolic blood pressure + HDL-cholesterol + log(triglycerides) + glucose [*n* = 4270].

a P-values from Cox multivariable linear regression models including SPPB score in models in continuous form.

b Crude incidence rate per 1000 person-years

c Data are hazard ratio (95% confidence interval)

d Missing covariate data imputed with multiple imputation using chained equations (MICE) with *mice* package in R [*n* = 5325].

### Effect modification of SPPB-incident acute decompensated hospitalized heart failure associations

The multivariable-adjusted HR (95% CI) for the association of a 3-unit decrement in SPPB, equivalent to one interquartile range, with incident heart failure in the overall cohort was 1.41 (1.23–1.62, Table 3). The results of stratified analyses suggested that lower SPPB was associated with a higher risk of heart failure in every cohort subgroup investigated, except for Hispanic/Latina women and those without hypertension. Increased risks were consistent and of similar magnitude in all subgroups (*P*-interaction>0.10, all), except by age for which there was a statistically significant interaction (*P*-interaction = 0.096) albeit with modest differences in HRs (<80 years: HR = 1.45, 95% CI = 1.09–1.92; ≥80 years: HR = 1.40, 95% CI = 1.20–1.63) and for the Reynold's Risk Score for which a statistically significant interaction (*p* = 0.004) was observed albeit for a modest difference in HRs when comparing those with below median RRS (<9.9: HR = 1.48, 95% CI = 1.12–1.95) to those with above median RRS (≥9.9: HR = 1.37, 95% CI = 1.14–1.64). Effect modification results using imputed data were consistent in direction and comparable in magnitude with the complete case analysis (Supplementary Table S3).

**Table 3:** Associations of a One-Interquartile Range (3-Unit) Decrement in SPPB with Incident Acute Decompensated Hospitalized Heart Failure Stratified by Selected Baseline Characteristics.

	*N*	No. Events	HR (95% CI)^a^	p-interaction
Total Sample	5253	302	1.41 (1.23–1.62)	
**Age**				0.096
< 80 Years	2681	83	1.45 (1.09–1.92)	
≥ 80 Years	2572	219	1.40 (1.20–1.63)	
**BMI**				0.749
< 30 kg/m^2^	3613	208	1.41 (1.20–1.66)	
≥ 30 kg/m^2^	1640	94	1.41 (1.10–1.80)	
Reynolds Risk Score*				0.004
< 9.9	2129	83	1.48 (1.12–1.95)	
≥ 9.9	2144	169	1.37 (1.14–1.64)	
**MVPA**				0.119
< 45 min/day	2621	215	1.29 (1.10–1.52)	
≥ 45 min/day	2632	87	1.60 (1.23–2.09)	
**Diabetes**				0.633
Without	4181	217	1.44 (1.23–1.69)	
With	1072	85	1.32 (1.02–1.71)	
**Hypertension**				0.890
Without	1498	61	1.30 (0.96–1.76)	
With	3755	241	1.45 (1.25–1.69)	
**Race/Ethnicity**				0.513
White	2550	202	1.43 (1.22–1.68)	
Black	1784	75	1.50 (1.12–2.01)	
Hispanic	919	25	1.03 (0.61–1.72)	

Abbreviations: HR = hazard ratio; CI = confidence interval; BMI = body mass index; MVPA = moderate-to-vigorous physical activity.

Reynolds risk score and MVPA were split at the median.

a Model 3 (Table 2) was used for all hazard ratios; adjusted for age, race/ethnicity (except for race/ethnicity strata), education, smoking status, alcohol use, diabetes (except for diabetes strata), hypertension (except for hypertension strata), COPD, osteoarthritis, depression, and BMI.

⁎ 10-Year predicted probability (%) of a clinical CVD event.

### Sensitivity analyses

Results were similar to those in the primary analysis after excluding 383 cases of prevalent MI or stroke at baseline, and after excluding 18 cases of acute decompensated hospitalized heart failure that occurred within the first six months (Supplementary Tables S1 and S2). The HR (95% CI) for a one-unit decrement in each SPPB component test (model 3 covariates) was 1.30 (1.17–1.43) for the chair stand test score, 1.17 (1.06–1.28) for the balance test score, and 1.11 (1.00–1.22) for the gait speed score. When all component tests were included in the same multivariable model, HRs for chair stand, balance, and gait speed were 1.27 (1.14–1.41), 1.11 (1.01–1.22), and 1.01 (0.90–1.12), respectively.

## Discussion

In this racially/ethnically diverse prospective cohort of older women, lower SPPB was associated with higher risk of incident acute decompensated hospitalized heart failure, independent of several relevant covariates including accelerometer-measured total sedentary time and MVPA, measured systolic blood pressure, treated hypertension and diabetes, and other cardiovascular biomarkers. Compared to women with high SPPB, the multivariable-adjusted risk of acute decompensated hospitalized heart failure for those with very low, low, and moderate SPPB was 2.22, 1.63, and 1.39-fold higher, respectively. Stratification over cohort subgroups showed general consistency of the direction and strength of associations, particularly for women with baseline obesity, diabetes, or hypertension. In the larger WHI, a sizeable proportion of incident heart failure is attributed to these three factors [20]. We show clear evidence that low physical function defined by the SPPB score is significantly associated with excess risk of acute decompensated hospitalized heart failure in high-risk cohort subgroups due to existing obesity (BMI ≥30; HR = 1.41), diabetes (HR = 1.32), or hypertension (HR = 1.45). Overall, the implication of this study is that low physical function, assessed by the SPPB, is strongly linked with development of heart failure in later life and, thus, might be useful in clinical settings when evaluating patient's heart failure risk.

The observed significant inverse and linear dose-response association between SPPB and incident heart failure suggests that enhanced physical function could contribute to heart failure prevention across a wide range of SPPB scores in older ambulatory women. This is a highly relevant observation for an aging US population. Physical function, as measured by SPPB and other performance tests, declines over time in older adults [21]. Physical function limitations affect a high proportion of adults 60 years and older, where heart failure burden is also substantial [22]. Thus, approaches to screen for low physical functioning among older adults in clinical settings as well as efforts to promote maintenance of adequate physical function in later life at the population level could be critical components of heart failure risk stratification and prevention with aging.

The results of the present study extend the existing literature on the associations of SPPB scores with aging-related outcomes. A meta-analysis of findings from published observational studies resulted in a summary relative risk for all-cause mortality of 3.25, 2.14, and 1.50 when comparing SPPB scores of 0-3, 4–6, and 7–9, respectively, with 10–12 (*P* < 0.001, all) [5]. Low performance on individual physical function tests, such as gait speed and hand grip strength were associated with higher risks of both all-cause and CVD mortality [23]. Limited published findings are available for SPPB scores and incident CVD in older adults. In a previous study we conducted among OPACH women, there was a significant inverse SPPB score-CVD association when comparing women with SPPB scores of 0–3 to those with SPPB scores of 10–12 (HR = 2.28, 95% CI: 1.50–3.48) [6].

Limited published data are available on SPPB scores and heart failure incidence. In the Health, Aging, and Body Composition (Health ABC) study on adults aged 70–79 at enrollment, a one-standard deviation decrement in their modified SPPB score was associated with higher risk of heart failure (HR = 1.24, 95% CI=1.13–1.36) [7], similar in magnitude to results in the present study for a one-standard deviation decrement in SPPB score (HR = 1.27, 95% CI = 1.12–1.44). Although we observed a statistically significant interaction on heart failure risk between SPPB and Reynold's Risk Score, the difference in HRs was modest when stratifying the sample using a median split. Future studies of this potentially complex interaction are needed. Collectively, results from the Health ABC study and our present study provide compelling evidence that low physical functioning, as measured by the SPPB, is a significant predictor of future heart failure development in older adults and could be an important factor in the context of both clinical risk assessment and primary prevention.

In the present study, the SPPB-heart failure association was independent of objective measures of physical activity and sedentary behavior. This suggests the involvement of additional mechanisms besides those for physical activity and sedentary behavior, namely accelerated aging [24,25]. The aging-related decline in skeletal muscle quality, function, and strength due to adipose accumulation in and around skeletal muscle, termed myosteatosis, is strongly associated with mortality and implicated in sarcopenia, frailty, and heart failure. [26], [27], [28]. The associations of poor physical function with sarcopenia, and of both sarcopenia and physical function with heart failure, suggest shared underlying pathogenic pathways [29], [30], [31]. Low SPPB scores could reflect the accumulation of deficits and loss of resiliency and function in multiple biological systems across organs such as skeletal muscle and cardiac tissue, which in part define sarcopenia and frailty [32,33]. Sarcopenia and frailty are strongly associated with poor clinical outcomes, and prevalence in older adults has been estimated at 34% and 50%, respectively [34], [35], [36], [37]. The results of the present study suggest that low physical functioning, as measured by the SPPB, might identify older adults already on a trajectory of functional loss predisposing to higher risk of heart failure. The stronger association observed for chair stands in the multivariate analysis of all three SPPB components suggests that loss of muscle strength could play a particularly important role in predisposing to heart failure.

Risk-based strategies for heart failure prevention and management have been identified as a key to reducing the burden of heart failure in the coming decades [38]. Critical to this effort will be sensitive and effective methods for heart failure risk stratification to guide primary prevention initiatives and to implement risk-based clinical trials. In a recent review on available office-based approaches for heart failure risk stratification, none of the algorithms included a measure of physical function [38]. The extent to which individual-level heart failure risk prediction algorithms are improved by addition of the SPPB score should now be evaluated.

Our study has several notable strengths. OPACH is a large and racially/ethnically diverse cohort of ambulatory older women, an understudied population with regards to heart failure epidemiology. Extensive health information collected at baseline allowed for detailed evaluation of relevant covariates as potential confounders and effect modifiers. The analysis of adjudicated acute decompensated hospitalized heart failure outcomes available in OPACH reduces the likelihood of bias due to outcome misclassification. Objective measures of physical activity and sedentary behavior were available, which is novel in epidemiological studies on physical functioning and heart failure. We carried out sensitivity analyses that excluded women with existing heart disease or stroke and heart failure cases identified early in follow-up, producing findings consistent with the primary results, reducing the likelihood that results are attributable to reverse causation. Additionally, we addressed missing data by using MICE to reduce selection bias and enhance the precision of study results. There are limitations that should be noted when considering the results of this study. The present study was unable to distinguish between heart failure subtypes defined by ejection fraction. The results of this study may not generalize to men or younger women as WHI focused on investigating determinants of morbidity in aging postmenopausal women. Only a single measurement of SPPB was available, precluding investigation of the associations of longitudinal changes in physical function with heart failure. Other measures, such as the 6-minute walk test (6MWT) have been used to quantify physical functioning levels of older adults. Distance covered during the 6MWT is strongly positively correlated (*r* = 0.76) with the SPPB summary score in older adults [39]. Lower scores on both performance tests are associated with higher risk of clinical outcomes in heart failure patients [40]. While both the SPPB and 6MWT are performance-based assessments of physical functioning, they reflect different dimensions of functional ability, for example walking endurance in the 6MWT and balance in the SPPB [41]. The extent to which associations with incident heart failure differ among measures of physical functioning requires further investigation.

The SPPB can be implemented in clinical settings with relatively low administrative burden and could provide useful prognostic information to healthcare providers of older adults beyond heart failure risk assessment [5,6]. Physical functioning can be improved in older adults by increasing physical activity including strength and aerobic training, suggesting that interventions aimed at preserving physical function could slow aging and reduce heart failure risk in later life [42]. In addition, older women with low SPPB scores may benefit from CVD prevention and HF surveillance.

## Declaration of Competing Interests

The authors declare that they have no known competing financial interests or personal relationships that could have appeared to influence the work reported in this paper.

## Appendix Supplementary materials

Image, application 1
