# Supplementary material for: The short physical performance battery and incident heart failure among older women: the OPACH study
Source: Am J Prev Cardiol. 2021 Aug 20;8:100247. doi: 10.1016/j.ajpc.2021.100247 (PMC8441145; doi:10.1016/j.ajpc.2021.100247)
Supplement: Supplementary file 1 [file mmc1.docx]

**Supplemental material for SPPB & HF manuscript**

**Supplementary Table S1**. Associations of Short Physical Performance Battery (SPPB) Categories with Incident Acute Decompensated Hospitalized Heart Failure in OPACH Women after Excluding Data from 383 Cases of Prevalent Myocardial Infarction and Stroke (n=4942).

|  | SPPB Categories | | | |  |
| --- | --- | --- | --- | --- | --- |
|  | Very Low  (SPPB: 0-3) | Low (SPPB: 4-6) | Moderate (SPPB: 7-9) | High (SPPB: 10-12) | p-trend^a^ |
|  | n=237 | n=900 | n=2139 | n=1767 |  |
| Events [rate^b^] | 28 [24.4] | 70 [14.0] | 105 [8.2] | 56 [5.0] | <0.001 |
| Model 1^c^ | 3.17 (1.99-5.06) | 2.12 (1.48-3.04) | 1.44 (1.04-2.00) | 1 (ref) | <0.001 |
| Model 2^c^ | 2.77 (1.72-4.46) | 1.95 (1.35-2.81) | 1.38 (0.99-1.91) | 1 (ref) | <0.001 |
| Model 3^c^ | 2.67 (1.65-4.34) | 1.92 (1.32-2.78) | 1.36 (0.97-1.89) | 1 (ref) | <0.001 |
| Model 4^c^ | 2.28 (1.39-3.73) | 1.69 (1.16-2.46) | 1.26 (0.90-1.76) | 1 (ref) | <0.001 |
| Model 5A^c^ | 2.23 (1.30-3.84) | 1.71 (1.14-2.56) | 1.30 (0.91-1.85) | 1 (ref) | <0.001 |
| Model 5B^d^ | 2.13 (1.30-3.50) | 1.65 (1.13-2.40) | 1.23 (0.88-1.72) | 1 (ref) | <0.001 |

| ^a^ P-values from Cox multivariable linear regression models including SPPB score in models in continuous form. |
| --- |
| ^b^ Crude incidence rate per 1000 person-years |
| ^c^ Data are hazard ratio (95% confidence interval) |
| Model 1 is age and race/ethnicity adjusted [n=4942].  Model 2 = Model 1 + education + smoking status + alcohol use + diabetes + hypertension + COPD + osteoarthritis + depression [n=4910].  Model 3 = Model 2 + BMI [n=4876].  Model 4 = Model 3 + sedentary time + moderate-to-vigorous physical activity [n=4876].  Model 5 = Model 4 + systolic blood pressure + HDL-cholesterol + log(triglycerides) + glucose [n=3975]. |

^d^ Missing covariates imputed with multiple imputation using chained equations (MICE) with *mice* package in R.

**Supplementary Table S2**. Associations of Short Physical Performance Battery (SPPB) Categories with Incident Heart Acute Decompensated Hospitalized Failure in OPACH Women After Excluding Data from 18 Heart Failure Cases Identified Within the First 6 Months of Follow-Up (n=5307).

|  | SPPB Categories | | | |  |
| --- | --- | --- | --- | --- | --- |
|  | Very Low  (SPPB: 0-3) | Low (SPPB: 4-6) | Moderate (SPPB: 7-9) | High (SPPB: 10-12) | p-trend^a^ |
|  | n=256 | n=949 | n=2268 | n=1834 |  |
| Events [rate^b^] | 30 [23.0] | 73 [13.4] | 123 [8.9] | 62 [5.3] |  |
| Model 1^c^ | 2.96 (1.89-4.64) | 1.95 (1.38-2.75) | 1.51 (1.11-2.05) | 1 (ref) | <0.001 |
| Model 2^c^ | 2.54 (1.61-4.02) | 1.76 (1.23-2.50) | 1.43 (1.05-1.95) | 1 (ref) | <0.001 |
| Model 3^c^ | 2.49 (1.57-3.96) | 1.75 (1.22-2.49) | 1.42 (1.04-1.94) | 1 (ref) | <0.001 |
| Model 4^c^ | 2.12 (1.32-3.40) | 1.54 (1.08-2.21) | 1.32 (0.96-1.81) | 1 (ref) | <0.001 |
| Model 5A^c^ | 2.03 (1.20-3.43) | 1.57 (1.06-2.33) | 1.42 (1.01-1.99) | 1 (ref) | 0.001 |
| Model 5B^d^ | 2.01 (1.25-3.23) | 1.51 (1.05-2.16) | 1.30 (0.95-1.78) | 1 (ref) | <0.001 |

| ^a^ P-values from Cox multivariable linear regression models including SPPB score in models in continuous form. |
| --- |
| ^b^ Crude incidence rate per 1000 person-years |
| ^c^ Data are hazard ratio (95% confidence interval) |
| Model 1 is age and race/ethnicity adjusted [n=5307].  Model 2 = Model 1 + education + smoking status + alcohol use + diabetes + hypertension + COPD + osteoarthritis + depression [n=5274].  Model 3 = Model 2 + BMI [n=5235].  Model 4 = Model 3 + sedentary time + moderate-to-vigorous physical activity [n=5235].  Model 5 = Model 4 + systolic blood pressure + HDL-cholesterol + log(triglycerides) + glucose [n=4259]. |

^d^ Missing covariate data imputed with multiple imputation using chained equations (MICE) with *mice* package in R.

**Supplementary Table S3**. Associations of a One-Interquartile Range (3-Unit) Decrement in SPPB with Incident Acute Decompensated Hospitalized Heart Failure Stratified by Selected Baseline Characteristics Using Imputed Data.

|  | n | No. Events | HR (95% CI)^a^ | p-interaction |
| --- | --- | --- | --- | --- |
| Total Sample | 5325 | 306 | 1.39 (1.21-1.59) |  |
| **Age** |  |  |  | 0.119 |
| < 80 Years | 2717 | 84 | 1.41 (1.06-1.88) |  |
| ≥ 80 Years | 2608 | 222 | 1.38 (1.19-1.61) |  |
| **BMI** |  |  |  | 0.714 |
| < 30 kg/m2 | 3663 | 212 | 1.38 (1.17-1.63) |  |
| ≥ 30 kg/m2 | 1662 | 94 | 1.40 (1.09-1.80) |  |
| **Reynolds Risk Score*** |  |  |  | 0.013 |
| < 9.9 | 2705 | 98 | 1.44 (1.11-1.88) |  |
| ≥ 9.9 | 2620 | 208 | 1.37 (1.17-1.62) |  |
| **MVPA** |  |  |  | 0.169 |
| < 45 minutes/day | 2662 | 217 | 1.28 (1.09-1.50) |  |
| ≥ 45 minutes/day | 2663 | 89 | 1.55 (1.19-2.03) |  |
| **Diabetes** |  |  |  | 0.741 |
| Without | 4242 | 221 | 1.41 (1.20-1.65) |  |
| With | 1083 | 85 | 1.32 (1.02-1.72) |  |
| **Hypertension** |  |  |  | 0.866 |
| Without | 1521 | 62 | 1.28 (0.94-1.75) |  |
| With | 3804 | 244 | 1.32 (1.23-1.66) |  |
| **Race/Ethnicity** |  |  |  | 0.474 |
| White | 2587 | 206 | 1.40 (1.20-1.65) |  |
| Black | 1806 | 75 | 1.48 (1.10-2.00) |  |
| Hispanic | 932 | 25 | 1.02 (0.56-1.83) |  |

Abbreviations: HR = hazard ratio; CI = confidence interval; BMI = body mass index; MVPA = moderate-to-vigorous physical activity.

^a^ Model 3 (Table 2) was used for all hazard ratios; adjusted for age, race/ethnicity (except for race/ethnicity strata), education, smoking status, alcohol use, diabetes (except for diabetes strata), hypertension (except for hypertension strata), COPD, osteoarthritis, depression, and BMI.

Reynolds risk score and MVPA were split at the median.

*10-Year predicted probability (%) of a clinical CVD event
